# The web-based multiplex PCR primer design software Ultiplex and the associated experimental workflow: up to 100- plex multiplicity

**DOI:** 10.1186/s12864-021-08149-1

**Published:** 2021-11-18

**Authors:** Jie Yuan, Ji Yi, Meixiao Zhan, Qingqing Xie, Ting Ting Zhen, Jian Zhou, Zeqing Li, Zhou Li

**Affiliations:** 1 General Surgery Center, Zhujiang Hospital, Southern Medical University, 253 Industrial Boulevard, Guangzhou, 510280 Guangdong Province China; 2 Department of General Surgery, Foshan Fosun Chancheng Hospital, Foshan, 528010 China; 3 Medical Department, Wuhan Igenebook Biotechnology co., Ltd, Floor 3, building 1, Zone B, Gaonong Biological Park, 888 Gaoxin Avenue, Wuhan, 430014 Hubei Province China; 4 Zhuhai Interventional Medical Center, Zhuhai City People’s Hospital, Zhuhai, 519000 China; 5 College of Landscape Architecture, Central South University of Forestry and Technology, Changsha, 410004 China

**Keywords:** Primer design, Multiplex, Software, Web interface, Variant

## Abstract

**Background:**

A large number of variants have been employed in various medical applications, such as providing medication instructions, disease susceptibility testing, paternity testing, and tumour diagnosis. A high multiplicity PCR will outperform other technologies because of its lower cost, reaction time and sample consumption. To conduct a multiplex PCR with higher than 100 plex multiplicity, primers need to be carefully designed to avoid the formation of secondary structures and nonspecific amplification between primers, templates and products. Thus, a user-friendly, highly automated and highly user-defined web-based multiplex PCR primer design software is needed to minimize the work of primer design and experimental verification.

**Results:**

Ultiplex was developed as a free online multiplex primer design tool with a user-friendly web-based interface (http://ultiplex.igenebook.cn). To evaluate the performance of Ultiplex, 294 out of 295 (99.7%) target primers were successfully designed. A total of 275 targets produced qualified primers after primer filtration, and 271 of those targets were successfully clustered into one compatible PCR group and could be covered by 108 primers. The designed primer group stably detected the rs28934573(C > T) mutation at lower than a 0.25% mutation rate in a series of samples with different ratios of HCT-15 and HaCaT cell line DNA.

**Conclusion:**

Ultiplex is a web-based multiplex PCR primer tool that has several functions, including batch design and compatibility checking for the exclusion of mutual secondary structures and mutual false alignments across the whole genome. It offers flexible arguments for users to define their own references, primer Tm values, product lengths, plex numbers and tag oligos. With its user-friendly reports and web-based interface, Ultiplex will provide assistance for biological applications and research involving genomic variants.

**Supplementary Information:**

The online version contains supplementary material available at 10.1186/s12864-021-08149-1.

## Background

The relationship between phenotype and variations in the human genome has been progressively clarified. Over 88 million variants (84.7 million single-nucleotide polymorphisms (SNPs), 3.6 million short insertions/deletions (indels), and 60,000 structural variants) had been characterized as of 2015 [1]. The Online Mendelian Inheritance in Man (OMIM™) database had 13,005 entries on October 1, 2001 [2]. A large number of variants have been employed in various applications related to people’s lives, such as providing medication instructions, disease susceptibility testing, paternity testing, and tumour diagnosis. Hereditary nonpolyposis colorectal cancer syndrome (HNPCC) is the most common hereditary form of colorectal cancer. Patients with HNPCC exhibit an up to 80% increase in the lifetime risk of colorectal cancer and an up to 60% increase in the lifetime risk of endometrial cancer [3]. HNPCC results from a germline mutation in one of four mismatch repair (*MMR*) genes [4]. MMR genes and other hereditary tumour genes also contribute to primary hepatocellular carcinoma, breast cancer and other cancers [5]. Early detection of these hereditary gene mutations provides adequate time for tumour prevention and periodic examinations such as colonoscopy.

To detect hundreds of genome variants (such as hereditary tumour gene mutations), whole-genome sequencing and target capture sequencing are usually needed to cover such a large number of variants [6]. However, once the target number of multiplex PCRs is within the range of hundreds, PCR-based target sequencing will outperform these other technologies due to its lower cost, reaction time and sample consumption. For example, whole exon sequencing (WES), one kind of target capture sequencing method, which can cover 30–60 million bases of the human genome, usually costs $150 ~ 300 per sample in China, needs a 200 ng ~ 2 μg DNA sample and takes 2–3 days to complete library construction. Another method suitable for genome variant detection is HumanCytoSNP-12 chips (Illumina), which cover ~ 301,000 SNPs and other genetic markers. However, HumanCytoSNP-12 chips still cost $267 per sample in China, need a 200 ng DNA sample and take 3 days to complete. When interrogating less than 200 genomic variants, self-tailored multiplex PCR will be more suitable, economical and efficient. The cost of self-tailored multiplex PCR only relies on primer synthesis, PCR reagents, and sequencing, which usually costs approximately $15 ~ 30 in China. In addition, DNA sample consumption during multiplex PCR is less than 100 ng, and the library construction only takes half a day to complete.

Self-tailored multiplex PCR methods mainly depend on well-designed primers. However, open-source multiplex PCR primer design software is rarely applicable (Table 1), particularly at the 100-plex level. To conduct multiplex PCR, primers need to be carefully designed to avoid the formation of secondary structures and nonspecific amplification between primers, templates and products [7]. Primer3 is the most popular open-source group of programs, programming libraries and web interfaces for assisting researchers with PCR primer design [8]. However, these programs lack multiplex primer clustering and filtering functions. Primer-BLAST allows users to design new target-specific primers in a single step and to check the specificity of pre-existing primers [9]. However, it is only suitable for designing one primer pair at a time based on web interfaces. Other primer design software programs, such as Oligo 7 [10], PrimerSelect [11], Primer Premier [12], and MuPlex [13], are neither multiplex programs nor are they free for all users. More importantly, multiplex PCR primer design software that can eliminate nonspecific amplification of the whole genome in multiplex PCR is rare and usually requires strenuous experimental validation.

**Table 1:** Comparison of different primer design software programs

	Ultiplex	PrimerPlex	MPD	Primer3	Primer-BLAST	Oligo
Multiplex PCR	Yes	Yes	Yes	No	No	No
Input	BED	FASTA	BED	FASTA	FASTA	FASTA
Web interface	Yes	No	No	Yes	Yes	No
Free for academics	Yes	No	Yes	Yes	Yes	No
Avoid SNPs in primers	Yes	Yes	Yes	–	No	No
Avoid primers in repeat areas	Yes	No	Yes	No	No	No
Single primer secondary structure check	Yes	Yes	No	Yes	Yes	Yes
Specificity checking alignment algorithm in the whole genome	Yes	No	No	No	Yes	No
Mutually compatible without secondary structure	Yes	Yes	No	–	–	–
Mutual specificity checking alignment algorithm in the whole genome	Yes	No	No	–	–	–
Self-defined species genome	Yes	No	No	–	Yes	–
Next-generation sequence addition	Yes	Yes	Yes	–	–	–
Graphic overview of primers found	Yes	–	No	No	Yes	Yes

Here, we developed the web-based multiplex PCR primer design software “Ultiplex”. It was developed in the Python language with the Flask, Primer3 core and BLASTn+ command-line tools. Ultiplex provides the combined performance of Primer3 and BLASTn+ in a user-friendly interface on a high-performance computational platform. It not only designs primers but also evaluates the performance of different primer pairs in a single reaction and filters and clusters multiplex primers.

## Implementation

### Ultiplex-core program

**Fig. 1:**
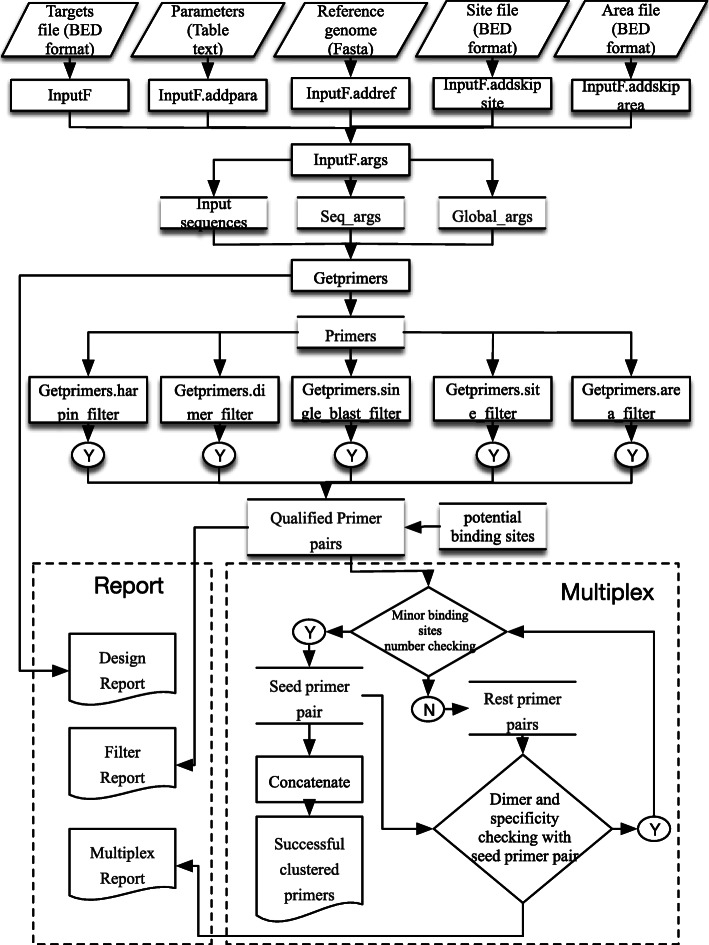
Diagram of the Ultiplex workflow

The main primer design and calculation procedures are conducted with the Ultiplex-core Program at the service centre, which was programmed in the Python language with the Flask, Primer3 core and BLAST+ command-line tools. The Ultiplex-core is formed by four modules (Fig. 1): “InputF” for argument input, “Getprimers” for primer design and filtration, “Multiplex” for multiplex primer pair clustering, and “Report” for the graphic overview of primer design.

**Fig. 2:**
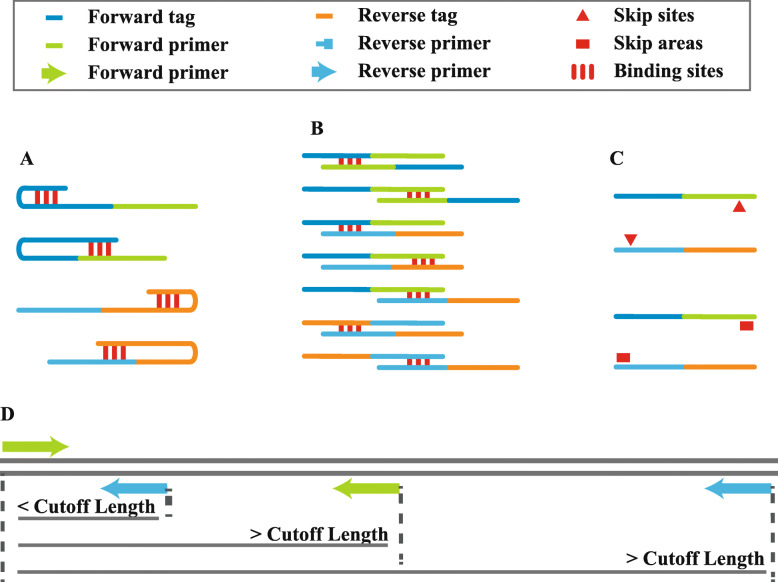
Filtration functions of Ultiplex. Primers need to be filtered with different functions to eliminate malfunctional pairs. **A** Getprimers.harpin_filter function. If there is a hairpin structure in the combined sequence of the primer and tag, the primer pair will be eliminated. **B** Getprimers.dimer_filter function. If there is a dimer structure between any two combined primer and tag sequences, the primer pair will be eliminated. **C** Getprimers.area_filter & Getprimers.site_filter functions. If any skipped sites or areas are located at the 3′ end of the primer, the primer pair will be eliminated. **D** Getprimers.single_blast_filter function. The potential binding sites of a single primer are evaluated, and if the amplicon length is below the cut-off value, the primer pair will be eliminated

1. “InputF” arguments input module: This module accepts the parameters from the web interface and generates the arguments and environment for subsequent modules. The genome reference is indexed with BLASTn+ command-line tools [14], and BLASTn+ database files are generated. The “pybedtools” package [15] is used to generate target sequences from genome references in the range of [target start position – product max size + 1, target end position + product max size]. Seq_args and Global_args, needed by the Primer3 core, are also generated, including Tm values, product size, primer size, GC% and primer numbers.
2. “Getprimers” primer design and filtration module: Primer pairs were designed with the primer-py package (https://github.com/libnano/primer3-py) and with target sequences and parameters from the previous module. The failed designed primers and reasons for failure were recorded. Secondary structures, such as hairpins and dimers, can affect primer amplification efficiency. Unlike other primer software, primers are individually checked for secondary structures. Under the “Getprimers.harpin_filter” function, primers and their 5′ tags are combined and tested with the “primer3.calcHairpin” function of primer-py, and primers showing hairpin secondary structures with Tm values over 45 °C are eliminated (Fig. 2A). Under the “Getprimers.dimer_filter” function, forward primers and reverse primers combined with their tags are compared to check dimer secondary structures with the “primer3.calcHeterodimer” function. Primers exhibiting dimer secondary structures whose Tm values are over 40 °C will be eliminated (Fig. 2B). Under the “Getprimers.area_filter” and “Getprimers.site_filter” functions, if the final 7 bp sequence at the 3′ end of a primer is located in a skipped site (such as SNPs and other user-defined sites) or repeat area (such as repeats, tandem repeats, indels and other user-defined areas), it will be filtered out (Fig. 2C).

More importantly, the “Getprimers.single_blast_filter” function is to check primer pair specificity. The specificity of the primer pair (or unique amplification of the genome) is checked by aligning forward/reverse primers to the whole genome with BLASTn+ command-line tools and calculating the possible amplicons of primer pairs within the genome. A single genome sequence will be assumed to be the potential binding site of a single primer if the following conditions are met. 1) The aligned genome sequence is longer than 12 bp, and the BLASTn+ e-value is over 1000. 2) The delta G value between the primer and the aligned genome site is above the threshold. 3) The mismatch at the 3′ end of each primer is smaller than 3 bp. 4) The mismatch number between the primer and aligned genome sites is smaller than 9 bp. If the distance between the potential binding site of the forward-reverse primer, forward-forward primer or reverse-reverse primer for one primer pair is below the threshold, the paired alignments will be assumed to be possible amplicons of the primers (Fig. 2D). When these possible amplicons are located outside of our target area, they will be assumed to be false-positive amplicons, and the related primer pairs will be filtered out. Throughout the process, the primer3-py package is used for delta G calculation.

**Fig. 3:**
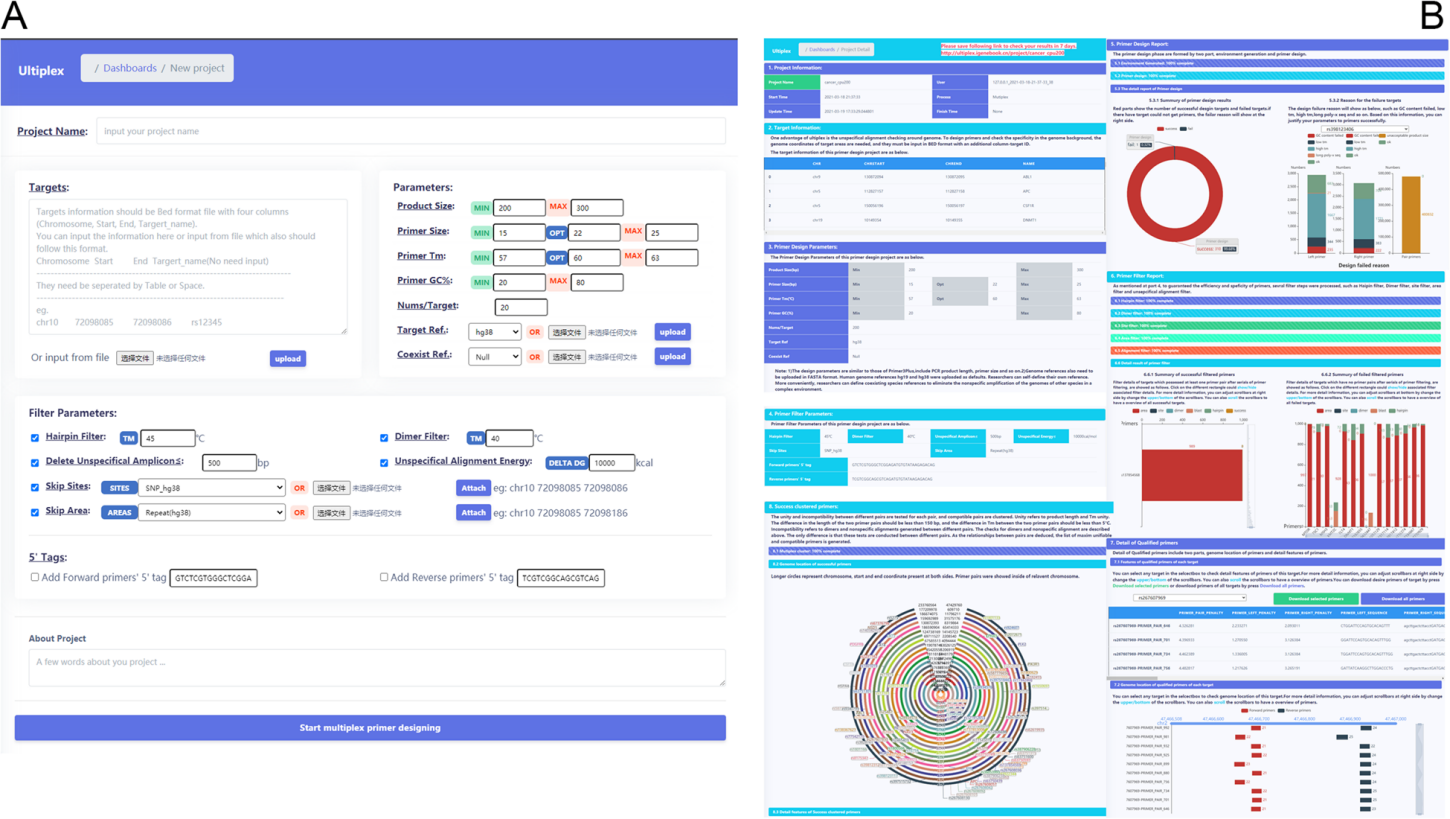
Web-based interface. **A** Project submission and parameter setting interface. Project information, primer parameters, filter parameters and tags can be input in this interface. **B** Project results interface. The detailed information and results of the submitted project

3) “Multiplex” for multiplex primer pair clustering: With the “Multiplex” function, the unity and incompatibility between different pairs are tested for each pair, and compatible pairs are clustered. Unity refers to product length and Tm unity. The difference in the length of the two primer pairs should be less than 150 bp, and the difference in Tm between the two primer pairs should be less than 5 °C. Incompatibility refers to dimers and nonspecific alignments generated between different pairs. The checks for dimers and nonspecific alignment are described above. The only difference is that these tests are conducted between different pairs. As the relationships between pairs are deduced, the list of maxim unifiable and compatible primers is generated.
4) “Report” graphic overview of primer design: The primer design, filtering and clustering results can be graphically illustrated by using “Report” functions, as can the failed targets and reasons for failure. The graphs are generated with the Python package pyecharts (https://github.com/pyecharts/pyecharts) and are illustrated for the user in the detailed project information interface (Fig. 3B).

### Tested variant list and primer design

We chose 250 hotspot mutations associated with HNPCC and hepatocellular carcinoma and 45 chemotherapy cytotoxicity-related variants (Table S1) as design targets to test Ultiplex. Primers (Table S2) were designed with Ultiplex following the provided guide.

### Cell collection and DNA extraction

HCT-15 and HaCaT cells were obtained from the Fifth Affiliated Hospital of Southern Medical University. The cells were centrifuged at 3000 g/min. DNA extraction was conducted with a Qiagen mini kit (Germany).

### Primer amplification and library construction

DNA was fragmented into pieces smaller than 500 bp with a Bioruptor® Pico instrument (Diagenode SA). The first round of PCR amplification was conducted with 10 μL of master mix (Vazyme, Nanjing, China), 1 μL of fragmented DNA (50 ng/μl), 1 μL of multiplex primer mix (the final concentration of each primer pair was 0.5 nM) and 8 μL of distilled deionized water (ddH2O). The first round of PCR amplification started at 94 °C for 5 min, followed by 15 cycles of 94 °C for 5 s, 60 °C for 30 s, 72 °C for 20 s, and then a final step at 72 °C for 5 min. The products were purified with AMPure XP beads (Agencourt, MA, USA). An 8 μL aliquot of the purified products was amplified with 10 μL of master mix (Vazyme, Nanjing, China), 2 μL of modified i5 and i7 primer mix (the final concentration of each primer was 10 nM) (Table S3). The thermal cycling program started at 94 °C for 30 s, followed by 12 cycles of 94 °C for 5 s, 60 °C for 30 s, and 72 °C for 20 s. The pooled products were purified with AMPure XP beads (Agencourt, MA, USA) and qualified on an Agilent 2100 Bioanalyzer (Agilent Technologies, Santa Clara, CA, USA) and a Qubit 3.0 Fluorometer (ThermoFisher Scientific, Waltham, MA, USA).

### Sequencing and analysis

The libraries were sequenced with a NovaSeq 6000 System (Illumina). The sequencing depth for each target was over 1000x. After sequencing was complete, raw data were assigned to each sample ID based on the barcode of the i5 and i7 primers. After low-quality read clearance by using fastp [16], clean reads were mapped to the reference sequence by using Bowtie 2 [17] with the parameter “--very-sensitive”. Only paired-end mapping reads were retained for SNP calling. SNP calling was conducted with SAMtools [18] and BCFtools [19].

## Results and discussion

### Web-based interface

Ultiplex was developed as an online multiplex primer design tool with a user-friendly web-based interface programmed with Flask, SQLite and Python software and other packages. There is no need for users to possess programming skills or construct a design environment to use Ultiplex, meaning that users (especially researchers) can easily design primer panels. The design parameters are similar to those of Primer3Plus [20]. There are differences in terms of the target input, reference file upload, skipped sites and areas defined and primer 5′ tags defined (Fig. 3A).

1) To design primers and check the specificity in the genome background, the genome coordinates of target areas are needed, and they must be input in BED format with an additional column-target ID. 2) Genome references also need to be uploaded in FASTA format. Human genome references hg19 and hg38 were uploaded as defaults. Researchers can self-define their own reference. More conveniently, researchers can define coexisting species references to eliminate the nonspecific amplification of the genomes of other species in a complex environment. 3) Researchers can define sites and areas that need to be skipped. There are two main categories of sequences that need to be masked: areas (repeats/tandem repeats/indels) and sites (SNPs and other small variances). PCR performance will be affected if primers are located in these areas. When primers are located in repeat areas, nonspecific amplification may occur. When the 3′ end of the primers is located in areas with a small amount of variance, PCR amplification of the variable sequences may fail. Additionally, users may need to exclude some areas due to their own needs. 4) For further analysis and sequencing of the product, sequencing primers need to be added to the 5′ ends of the library, and researchers can choose different strategies, such as “adapter ligation^”^ [21] or “two-step PCR” [22]. If researchers choose to use the “two-step PCR” strategy, 5′ tags need to be added at the 5′ end of the primers, and we provide one pair of tags for the Illumina sequencing platform as the default: GTCTCGTGGGCTCGGAGATGTGTATAAGAGACAG and TCGTCGGCAGCGTCAGATGTGTATAAGAGACAG. Researchers can define tags according to their own needs. Once parameters are submitted, a project is created and processed at our service centre. Progress will be updated as shown in Fig. 3B. With a simple click, detailed project information will be provided indicating the detailed design progress and completed design results.

### Comparison to other primer design tools

For primer design functions, Ultiplex offers several advanced functions that are not available in other software tools. Table 1 provides a brief summary of these functions, many of which are important for multiplex primer design. For example, Ultiplex is the only tool that offers the ability to examine the mutual compatibility of clustered multiplex primers in the whole genome. It is difficult to avoid nonspecific amplification when we simply mix the specified single primer pairs together because there may be false amplification between different pairs. The probability of nonspecific amplification will increase as PCR multiplicity increases. To avoid nonspecific amplification, Ultiplex can check the specificity between different primer pairs in the whole genome with BLASTn+ command-line tools and can check for secondary structures between different primer pairs. The compatible primer pairs will be clustered together, and primers for other targets will be redesigned or allocated into other clusters based on the user settings. Furthermore, users can define their own genome references and areas that they need to skip. This option is useful for microbe and pathogen researchers because their targets exist in complicated environments, including host and other microbe genomes. With Ultiplex, false amplification in other species can easily be avoided. Additionally, detailed design features, reasons for failure and cluster information are illustrated with graphic reports in Ultiplex.

For the principles behind primer design tools, BLASTn+ command-line tools and primer3 were reported to be used separately or combined for primer design, or even multiplex primer design in several studies. BatchPrimer3 was developed with Primer3 core and BLASTn+ for microsatellite (simple sequence repeat-SSR) and single nucleotide polymorphism marker primers [23]. However, BatchPrimer3 is mainly based on an allele-specific PCR strategy and cannot be used for multiplex primers. ThermoAlign is a tiled amplicon (long range sequence) resequencing primer design tool used in Primer3 core and BLASTn+, but it cannot be used for special sites or areas [24]. Yaheng Wang developed a multiplex PCR primer design system for targeted sequencing, which is similar to combining Ultiplex with Primer3-py and BLASTn+ [25]. However, this multiplex PCR primer design system lacks the ability to examine the mutual compatibility of clustered multiplex primers within the whole genome and is not available to all users.

### Thresholds of nonspecific alignments and secondary structures

**Fig. 4:**
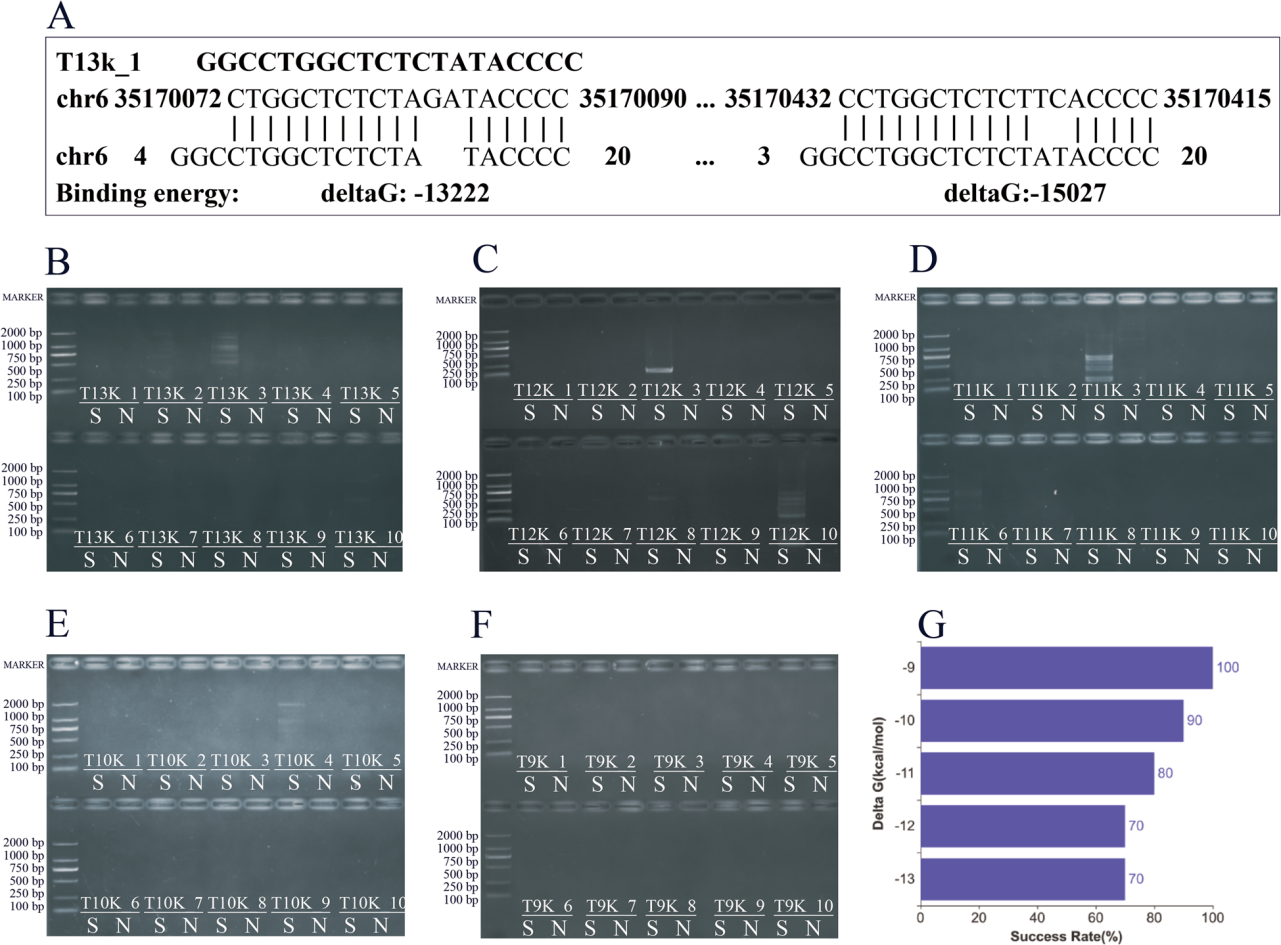
Performance of primer sets with different delta G cut-off values. **A** Genome alignment of a single primer, T13k_1. The nonspecific amplicon shows a strong binding stability on chr6. **B**, **C**, **D**, **E**, and **F** represent the amplification of the under − 13, − 12 ~ − 13, − 11 ~ − 12, − 10 ~ − 11, and over − 9 kcal/mol sets, respectively; S refers to the human genome DNA sample, and N refers to the water negative control. **G** Verification of primer sets with different delta G cut-off values

Hairpins, dimers and nonspecific alignments are three important parameters in the analysis of the specificity of single primer pairs and mutual primer compatibility. Tm and delta G are the main parameters used to determine whether harmful hairpins, dimers and nonspecific alignments may occur. We employed the nearest neighbour thermodynamic parameters of primer3 to calculate Tm and delta G. The default formulations of NaCl, Mg2+, DMSO, and dNTPs were described previously [26]. The Tm and delta G thresholds of hairpins, dimers and nonspecific alignments were set at 45 °C, 40 °C and − 10 kcal/mol, respectively, following a previous study [27]. Users are free to define thresholds for hairpins, dimers and nonspecific alignments before the design procedure. Five sets of single primers with different delta G cut-off values were tested. Each set of primers (Table 2) contained 10 single primers that represented the possible false amplicon at each delta G threshold (Fig. 4A). The accurate amplification rate (Fig. 4F and G) of primers with delta G values above − 10 kcal/mol was 100%. For primers with delta G values of − 10 ~ − 11, − 11 ~ − 12, − 12 ~ − 13 and under − 13 kcal/mol (Fig. 4B–F), the accurate amplification rates were 90, 70, 70, and 70%, respectively. This result shows that the default delta G threshold is sufficient to distinguish nonspecific alignments under our recommended PCR conditions.

**Table 2:** Delta G cut-off values for primer sets

deltaG cut-off	Primer names	Primer_sequences	Chr^*^	Length	Left_site deltaG^△^	Right_site deltaG^■^
<−13 k cal/mol	T13k_1	GGCCTGGCTCTCTATACCCC	6	325	− 13,223	−15,028
	T13k_2	ACGTTGCTCCCGCTCACC	16	945	−14,693	− 14,693
	T13k_3	AAGACCACGAGGCACAGC	20	1778	−13,580	− 13,372
	T13k_4	CCACAGCATCGTGACCCTG	2	1453	−15,972	−15,972
	T13k_5	AGCAAGGAAGTGGACAGGTC	8	572	−13,149	−13,149
	T13k_6	GTGTAGGAGCTGCGCCTC	5	503	−13,483	−13,420
	T13k_7	ATCTGCAGCAGTTTGGAGCC	6	742	−13,454	−13,454
	T13k_8	TCTCCACGATGCCGGCTG	9	1402	−13,003	−14,414
	T13k_9	GTTTCTTCTCCATCGCGGGG	17	1130	−13,847	−13,573
	T13k_10	CTCCATGGCTGATCTCCCCT	16	1736	−13,195	−13,195
−12 ~ − 13 k cal/mol	T12k_1	CAGATCACCTACAGCGCCA	11	1635	−12,176	−12,176
	T12k_2	TAACCCCCATAGCCCTCAAC	1	1645	−12,460	− 12,621
	T12k_3	CATATGGCCGTGTCTGGGG	2	239	−12,572	−12,021
	T12k_4	CCAGCCCAGCATTCTCCAG	19	1864	−14,841	−12,519
	T12k_5	GCAGCAGGGATTGGATGGC	15	1776	−12,218	−12,218
	T12k_6	GCAGGGATTGGATGGCCC	11	855	−12,436	−12,115
	T12k_7	CATCTTCGATTTGAGTGCCCC	12	1868	−12,150	−12,150
	T12k_8	TTCTTGAAGAGGACGGTGCC	2	1298	−12,580	−12,380
	T12k_9	TCTGAGCATTTGGGCTTGC	3	984	−12,562	−12,671
	T12k_10	CAAGATGTCGGGGAGTGGCC	17	1502	−12,976	−12,900
−11 ~ − 12 k cal/mol	T11k_1	GGCTAAGGGGGTACACTTCA	x	657	−11,112	−11,112
	T11k_2	CTTGGAAGTGGACGTAGGTGT	x	802	−11,333	−11,333
	T11k_3	GGTGTCTGTCCCGGCCAG	4	529	−13,905	− 11,348
	T11k_4	CATTTACAATCCAGCCAAATCACT	7	1475	−11,879	−11,879
	T11k_5	GCTCCTGGGGCATAGGGATG	3	335	−11,911	−11,911
	T11k_6	CAGGGATTGGATGGCCCC	11	516	−11,193	−11,193
	T11k_7	CTGGCTTGGCACTGGTCT	15	916	−11,926	−11,322
	T11k_8	AGCCCTGTGTGGTTCCAAG	6	916	−11,194	−11,175
	T11k_9	CTCACTCCATCCTGGACGT	2	452	−11,110	−11,110
	T11k_10	GCTCACTCCATCCTGGACGT	2	452	−11,868	−11,868
−10 ~ − 11 k cal/mol	T10k_1	CCTCCTTGTGCCCTAGAGTT	14	185	−10,271	−10,271
	T10k_2	cctttctgttgcctGTGGTAAG	5	161	−10,543	−10,224
	T10k_3	GCTTTTCCTCATGGGGCAAA	4	447	−10,575	−10,809
	T10k_4	CCAGGACAAGGCCTCATCC	4	325	− 10,242	− 10,853
	T10k_5	TTCCATGGCAGACTTGTAAGGA	14	401	−10,934	−10,934
	T10k_6	CAGTGTGGTAAAAACGTCAGGATG	2	464	−10,167	−10,167
	T10k_7	AGTTGCAGAACCTTGCCCT	14	375	−10,812	−10,350
	T10k_8	TCGAGACGGGGGTAAAGGAG	X	147	−10,799	−10,073
	T10k_9	AGGAATTCATGCCTTTGGGACA	6	191	−10,203	−10,678
	T10k_10	CTGCTCCATGGCTGATCTCC	4	316	−10,235	−10,809
−9 ~ − 10 k cal/mol	T9k_1	ATGGAGGCTGGATAGGAGGT	1	162	− 9516	− 9084
	T9k_2	GCCTCCTTGTGCCCTAGAGT	14	187	− 9598	−9598
	T9k_3	GGGTCTTGGCAAGTGGTACTG	6	394	− 9068	− 9307
	T9k_4	GCTCCTGGGGCATAGGGAT	3	337	− 9737	−9737
	T9k_5	GATTTCAGGGTGGTCACAGT	2	419	− 9299	− 9050
	T9k_6	ACGCTGCTATGGTTCTACAG	17	276	− 9320	− 9506
	T9k_7	GCAGCAGGGACTCACCATTG	2	409	− 9396	− 9861
	T9k_8	ATTCAGCGTGATGTGTAATGAGG	9	428	− 9135	− 9334
	T9k_9	AGAGGCTCCTGCAATTGACAG	9	302	− 9394	− 9330
	T9k_10	TCAGACCAGTGCTACCTGAGAG	9	202	− 9438	− 9577

*: Chromosome number, △: Delta G value (cal/mol) of Left aligned site of the primer on the possible false amplicon, ■: Delta G value (cal/mol) of right aligned site of the primer on the possible false amplicon

### Performance and results of primer design and single-target filtering

**Fig. 5:**
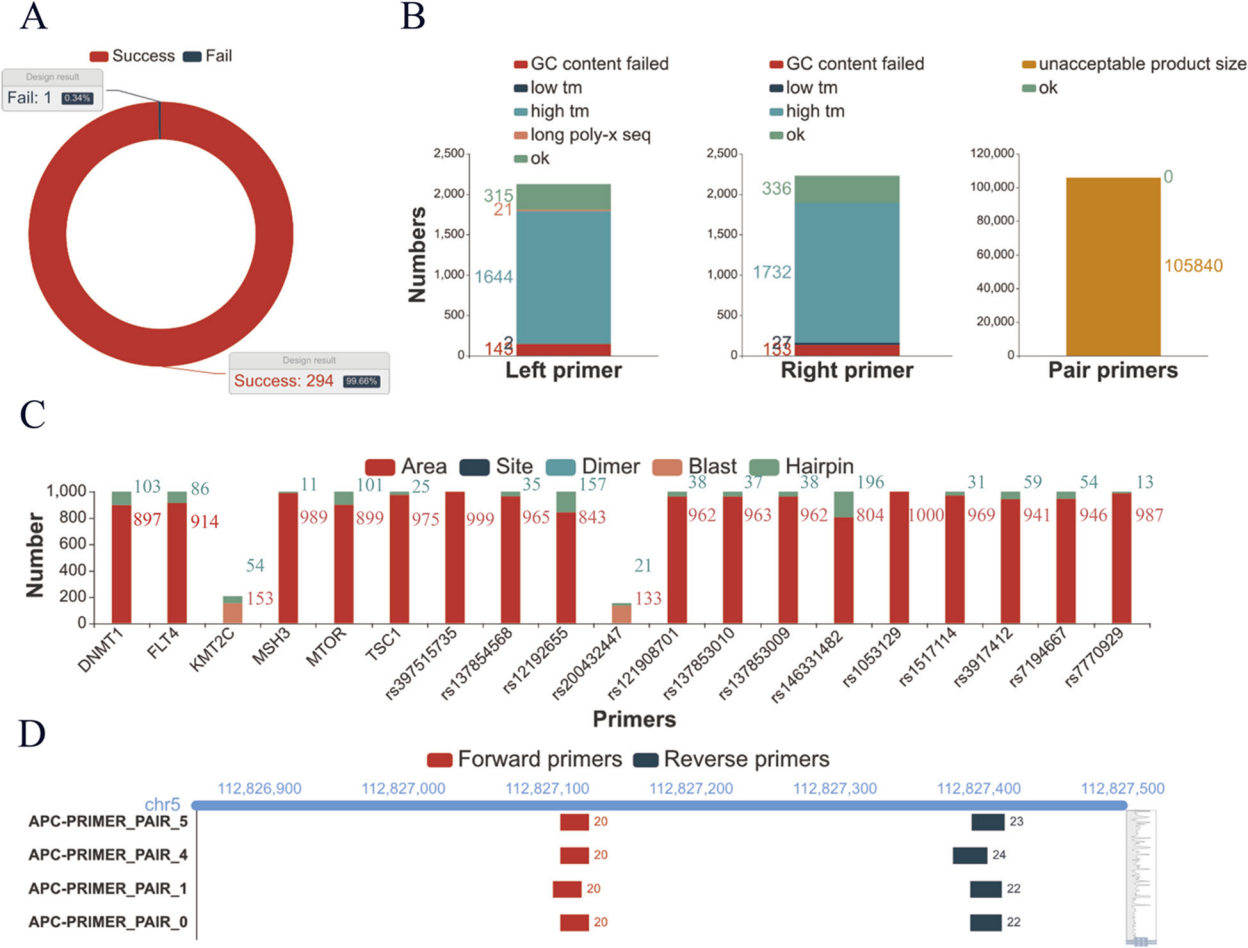
Performance and results of primer design and single-target filtering. **A** Summary of primer design results. **B** Reason for the failure of rsf398123406 primer design. **C** Reason for the failure of primer filtration. **D** Qualified primer pairs of APC targets

A total of 295 targets were designed with Ultiplex, among which 99.7% of sites were designed successfully, and 1 target (rs398123406) showed design failure (Fig. 5A). Product size is the main reason for failure. As shown in Fig. 5B, a total of 315 left primers and 336 right primers were designed successfully, but the left primers and right primers were not matched with each other according to the appropriate length. After checking the detailed input information, we found that the rs398123406 target was 450 bp in length (Table S1). An overly broad target range results in fewer choices for primer design. A narrower target range will correct the design result.

Nineteen of 294 successfully designed targets failed according to primer filtration (Fig. 5C). A location in a skipped area was the main cause of filtration, and 17 target primers located in such areas had to be skipped (these areas are actually repeat-sequence areas in the human genome according to our default setting). The other reasons for filtration were the identification of dimers or skipped sites and specificity in the genome. A total of 5.83% of the primer pairs were filtered out because of the specificity checks. The qualified primer pairs were generated and could be saved to local files with unique alignments (Fig. 5D, Table S5). Design and filtering information can be generated with the “Report” functions of Ultiplex. The resultant graphics have partial interaction functions and can provide to users to help them adjust their target areas and parameters during redesign based on information such as the identification of secondary structures, repeat areas, false alignments and so on.

### Multiplex primer clustering results

**Fig. 6:**
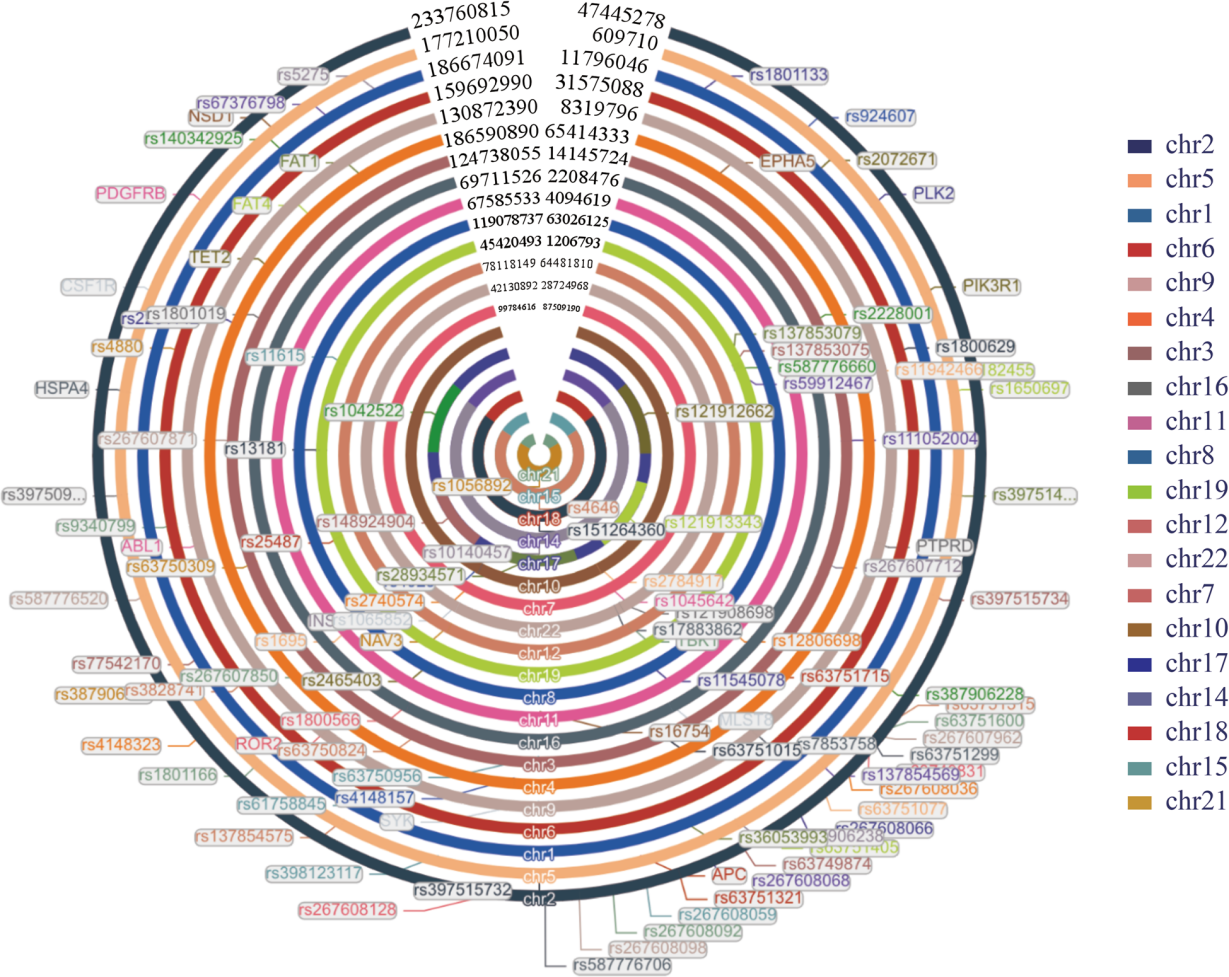
Locations of 108 multiplex primer pairs on the human genome. Circles refer to chromosomes on the human genome

**Fig. 7:**
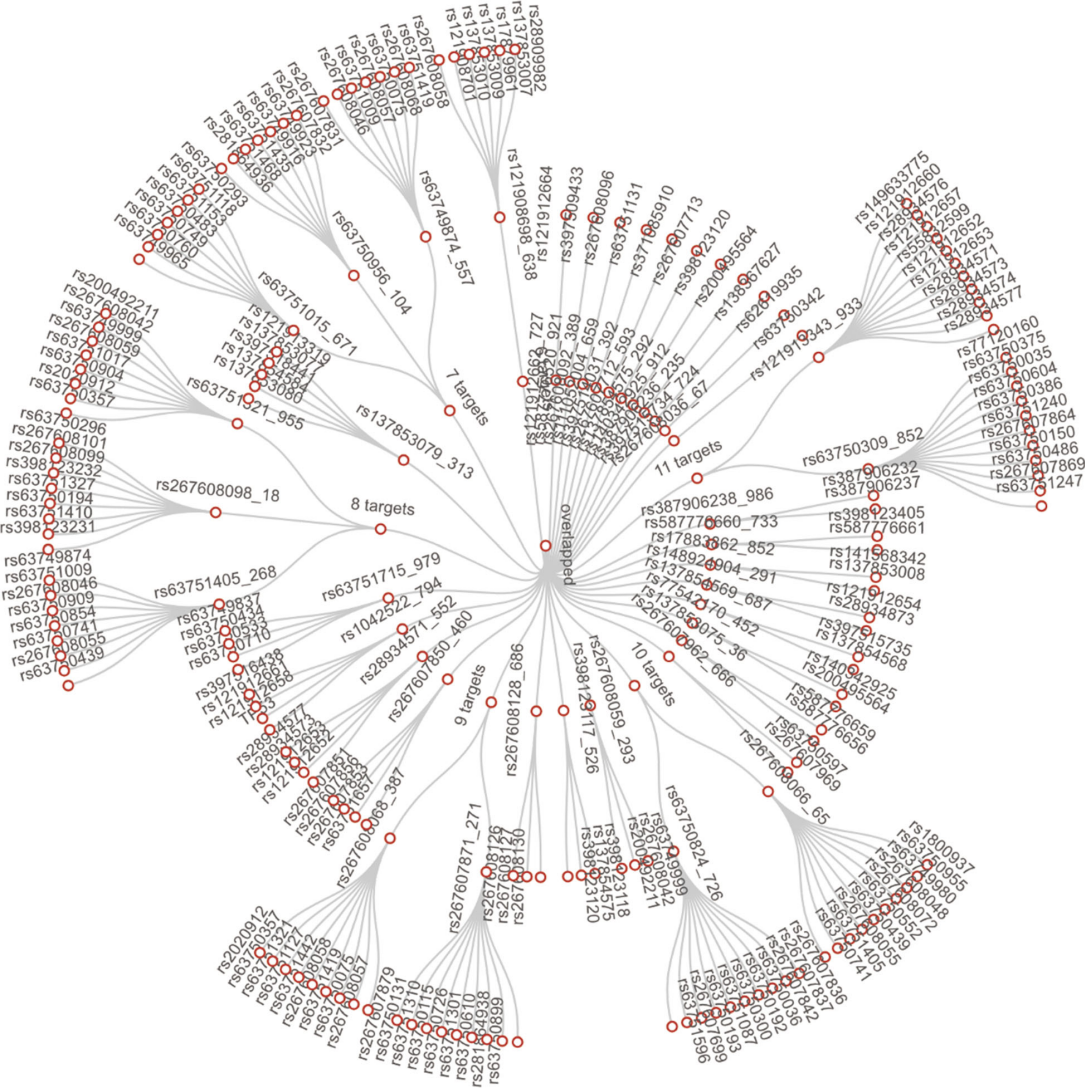
Summary of the overlap between successfully clustered sites

Among 275 targets, 261 were successfully clustered into one compatible PCR group. These 261 targets could be covered with 98 primer pairs due to the overlap between targets and primers (Fig. 6). Information on this overlap was also generated (Fig. 7). For example, rs267607850–460 overlapped with 4 targets (rs267607853, rs63751657 and 2 other targets), which means that these 4 targets were located in the primer pair rs267607850–460 amplicon. With the advantage of the single-nucleotide resolution of next generation sequencing (NGS), we could obtain polymorphism information not only for rs267607850 but also for the 4 other targeted polymorphisms. There was no need to design primers for these overlapping targets. Fourteen of 275 targets could not be integrated into this group because of secondary structures and false alignments, for which false alignment was the main reason.

**Fig. 8:**
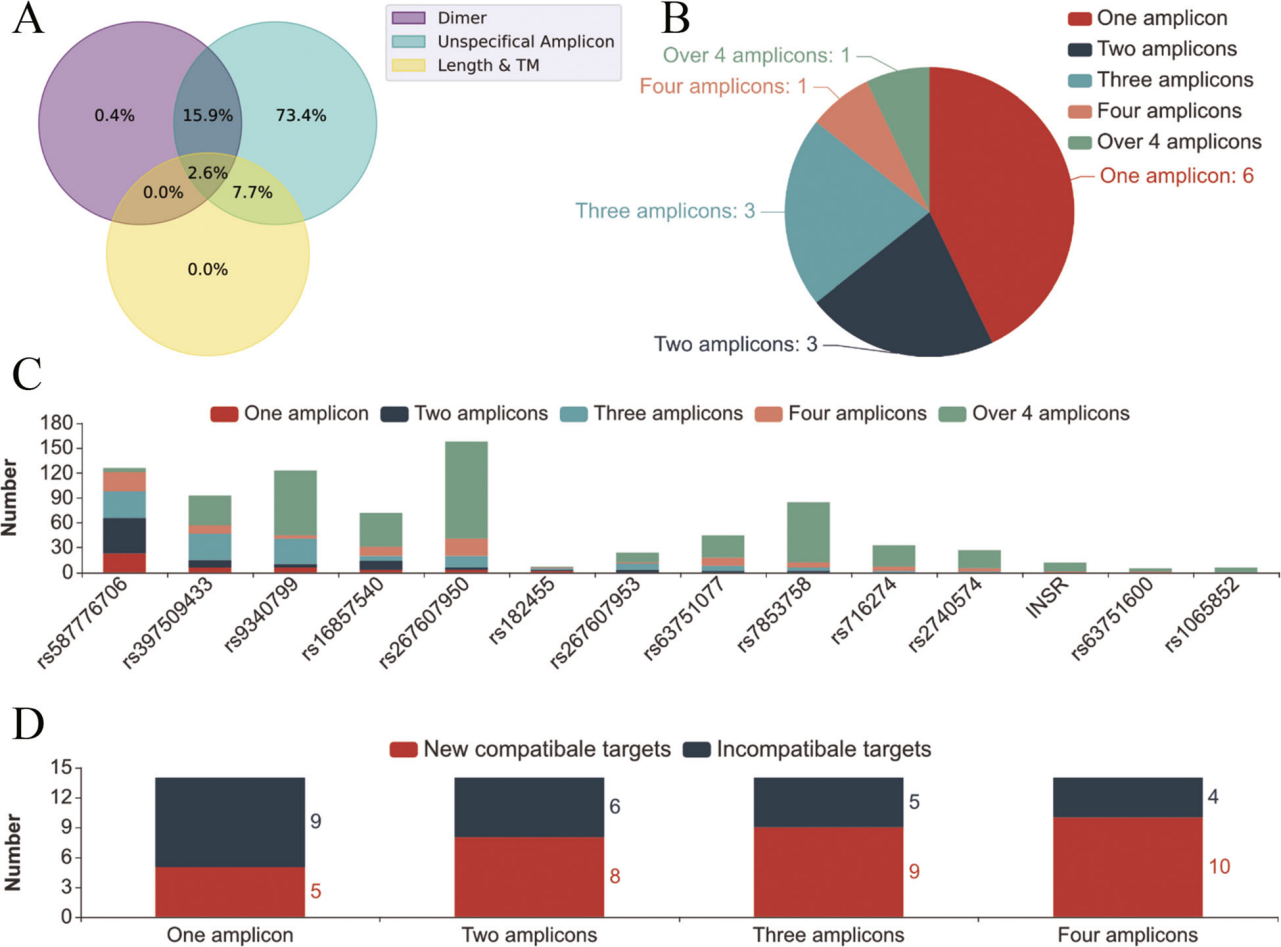
Multiplex primer clustering results. **A** The incompatibility between 14 failed target primers and 98 successfully clustered primers. **B** The number of false alignments between the 14 failed target primers and 98 successfully clustered primers. **C** The number of unspecific amplicon sites between incompatible targets and compatible group primers. **D** The number of new compatible targets increases as the number of unspecific amplicon site cut-off values increase when conduct multiplex compatible clustering

As shown in Fig. 8A, 99.6% of the primer pairs of targets that failed to be integrated into the compatible group showed false alignment with primer pairs in this compatible PCR group (Fig. 8B). Six of 14 targets possessed at least one primer pair that showed false alignment with only one primer pair in the compatible PCR group (Fig. 8B/C). The total target number of the compatible group increased as we lowered the threshold for the number of false alignments between primer pairs in this group (Fig. 8D). When we allowed there to be 1 false alignment between 14 incompatible target primers and the 98 compatible primer pairs, 5 pre-incompatible targets could be clustered in the compatible PCR group. When we allowed there to be 4 false alignments between those two groups, 10 pre-incompatible targets could be clustered in the compatible PCR group. As a result, 271 targets were included in a single primer cluster covered by 108 primer pairs (Fig. 6), and 4 targets (rs267607950, rs267607953, rs16857540, and rs716274) were incompatible with this cluster. Limited nonspecific amplification events are acceptable in next-generation sequencing PCRs, particularly for projects with a sufficient budget, because these amplicons can be distinguished clearly with next-generation sequencing and bioinformatic pipelines.

### Increased multiplex clustering speed and time and storage consumption

**Fig. 9:**
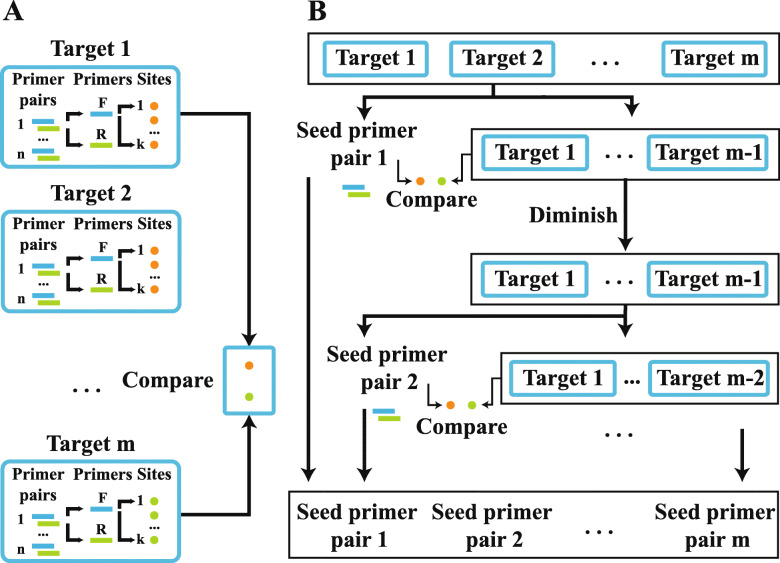
Cluster complexity of different strategies. **A** Normal contrast between every primer. **B** Seed primer pair strategy

Although multiplex cluster simulation could simplify tedious experimental library generation work and reduce the time requirement by months, the calculation process may be longer because of computer storage, CPU and argument settings. The multiplex clustering calculation step is the most memory-intensive and time-consuming calculation step in multiplex primer design, especially when the number of primers for each target is larger. When we needed to obtain a compatible relationship between m target primer pairs, we assumed that each target had n primer pairs and that both primers in one primer pair had k nonspecific sites on average; the multiplex cluster calculation was as follows: ${\complement}_m^2\times 4\times {\complement}_{n\times k}^1\times {\complement}_{n\times k}^1$ (Fig. 9A). To eliminate complexity and speed up the process, we assumed that one primer pair (seed primer pair), which possessed minimal nonspecific binding sites, showed the highest possibility of appearing in our ideal mutually compatible cluster. In each turn, one seed primer pair was selected from all primer pairs of all targets based on the number of nonspecific binding sites. The primer pairs for the remaining targets that were incompatible with this seed primer pair were deleted. The next seed primer pair was selected from the remaining primer pairs. After several iterations, when no primer pairs remained, the pool of all seed primer pairs was our ideal compatible multiplex primer cluster. Thus, the calculation procedure becomes much simpler (Fig. 9B).

After increasing the speed of the procedure, it was tested for time and computer storage consumption with different tasks. As shown in Table 3, when the initial primer number per target was two, Ultiplex required 1.3 h to design primers for 295 targets. When the initial primer number per target was increased to 100, the time required was 14.7 h. The complexity of the calculation process increases as the initial primer pair number (n) for one target or the target number (m) increases.

**Table 3:** Consumption of time and storage of 295 target primers

	2 primer pairs/target	20 primer pairs/target	50 primer pairs/target	100 primer pairs/target	200 primer pairs/target
Time	1.3 h	4 h	8 h	14.7 h	18 h
Memory	65G	65G	65G	65G	65G
CPU core	32	32	32	32	32
Multiplicity of cluster	56	85	97	98	103

### Multiplex PCR process and sequencing results

**Fig. 10:**
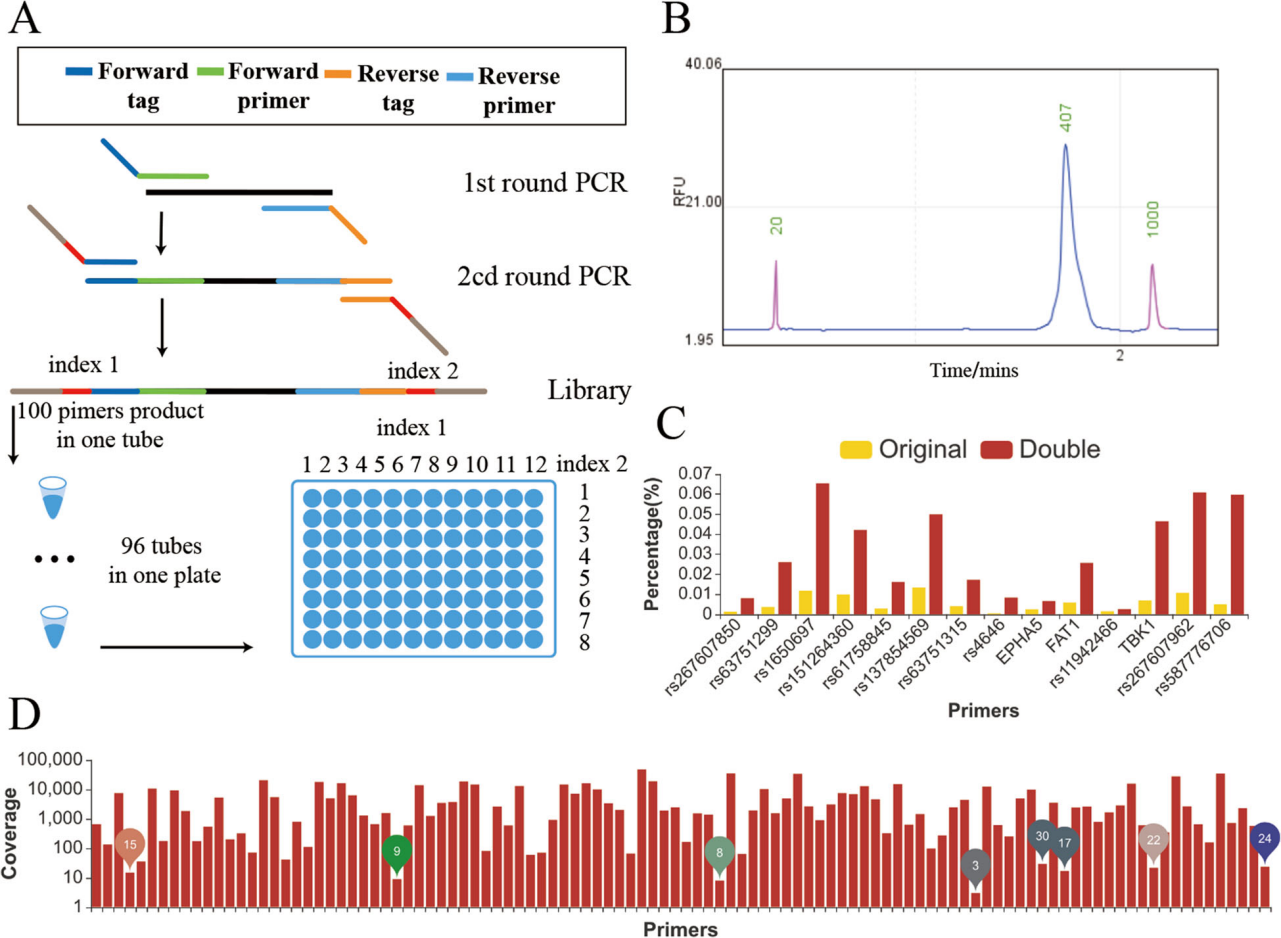
Multiplex PCR experimental process and sequencing results. **A** GT-seq experimental process. **B** Final library size. **C** The read coverage obtained with different primer concentrations. **D** Sequencing depth of targets

We modified the multiplex experimental strategy of Genotyping-in-Thousands by sequencing (GT-seq) with a previous template fragmentation step (Fig. 10A) to increase multiplicity. When we fragmented the DNA template to a size smaller than 500 bp, the multiplicity was increased from 68-plex to 98-plex. The improvement was caused by the eradication of nonspecific amplicons over 500 bp between multiple primer pairs. Rather than template fragmentation, altering the PCR extension time is the common strategy for controlling amplicon length and nonspecific amplicon length. For example, a 30-s PCR extension step with regular DNA Taq polymerase is predicted to amplify a 500 bp product and to reduce the generation of nonspecific amplicons longer than 2000 bp. However, due to the uncertainty of polymerase efficiency, this strategy cannot eradicate nonspecific amplicon production. With prior template fragmentation and our pairwise process for checking possible primer-genome amplicons smaller than 500 bp, 108-plex multiplex reactions can now be performed with high specificity.

A single primer pair showed a 93% amplification success rate (Fig. S1), and the multiplex PCR products were within the expected range (Fig. 10B). A total of 106 primer pairs could produce clear products, and 2 primer pairs produced faint minor products when used separately for the amplification of human DNA. A total of 108 primer pairs showed 100% specificity. These primers were specific to the human genome, as they generated unique products from human DNA. They also exhibited no secondary structures, such as dimers, as no products were generated from water. When the 108 primer pairs were pooled and amplified with a dedicated experimental workflow, the products were within our expected 200–500 bp range (Fig. 10B).

Two hundred megabase pairs of sequencing data per sample was generated with the 108 primer pairs. The average coverage of the 108 primer pairs was 5000X, and the alignment rate was 99.21%. Nonspecific amplicons were rarely generated, even though 10 nonspecific primer pairs were included in the reaction. Only 7 targets showed below 30X coverage (Fig. 10D), and as we doubled the concentration of the primers, the read coverage increased rapidly (Fig. 10C, Table S4). The performance of those low-efficiency primers in the multiplex PCR primer group could be enhanced by increasing the primer concentration.

### Sensitivity of variant detection

DNA from HCT-15 and HaCaT cell lines was analysed with the Ultiplex associated experimental and bioinformatic workflow. Information for 245 SNPs/variants was generated. In these 245 SNPs/variants, Rs28934573 of the *TP53* gene is significantly different between HCT-15 and HaCaT cell lines. This variant was tested to determine the detection limit and sensitivity of this system with a series of samples with different HCT-15 and HaCaT DNA ratios. The results show that multiplex PCR does not affect the sensitivity of single target detection. For rs28934573(C > T) (Table 4), this multiplex system stably detected mutation rates as low as 0.25%. These rates were 0.25, 0.29 and 0.19% for the simulated sample with a 0.25% theoretical value. The coefficient of variation was 20.68%. These results show that variant detection with the ‘Ultiplex’ multiplex primer group and associated experimental workflow is sensitive, stable and reproducible.

**Table 4:** Mutation rates of simulated samples of rs28934573 (C > T)

Mixture Ratio	Theoretical value	Mean	Repeat 1	Repeat 2	Repeat 3	Variance	CV
HCT-15	50.00%	50.64%	51.04%	49.52%	51.37%	0.99%	1.95%
HaCaT	0.00%	0.11%	0.13%	0.10%	0.10%	0.02%	15.75%
50% HCT-15/HaCaT	16.67%	17.42%	16.59%	17.80%	17.87%	0.72%	4.13%
25% HCT-15/HaCaT	10.00%	11.04%	11.86%	10.89%	10.37%	0.76%	6.85%
10% HCT-15/HaCaT	4.55%	5.34%	5.25%	5.75%	5.03%	0.37%	6.91%
5% HCT-15/HaCaT	2.38%	2.82%	2.50%	2.48%	3.47%	0.57%	20.09%
1% HCT-15/HaCaT	0.50%	0.63%	0.68%	0.37%	0.84%	0.24%	37.93%
0.5% HCT-15/HaCaT	0.25%	0.24%	0.25%	0.29%	0.19%	0.05%	20.68%
0.1% HCT-15/HaCaT	0.05%	0.16%	0.09%	0.30%	0.10%	0.12%	72.53%

## Conclusions

Ultiplex is a web-based multiplex PCR primer tool. With the associated experimental workflow, Ultiplex can be used to detect any genomic area with next generation sequencing for a species with a clear reference genome, including genotyping, disease associated variation detection, and species identification. Ultiplex has several functions, including batch design and compatibility checking for the exclusion of mutual secondary structures and mutual false alignments across the whole genome. It offers flexible arguments for users to define their own references, primer Tm values, product length, plex number and tag oligos. The input can be any BED file from any species reference. It can avoid repeat regions and variance sites at the end of primers to achieve precise amplification as well as those areas that users want to exclude. Finally, based on graphic primer design reports, users can not only design and generate highly specific multiplex PCR primers with Ultiplex but can also adjust their strategy to obtain the optimal multiplex primer clusters. The report displays a summary of the design, filtering, information clustering and redesign, and the detailed reasons for failure at any step. According to the report, the user is allowed to choose the generated primers or to change the strategy to generate a more specific primer set. With Ultiplex, we designed primers for 271 targets pooled in one reaction. The results showed that this primer set could achieve a 93% success rate. For genome mutation, this multiplex system stably detected 0.25% mutation rates. Providing user-friendly reports and web-based interface, Ultiplex will assist in biological applications and research involving genomic variants.

## Availability and requirements

**Project name:** Ultiplex

**Project home page:** http://ultiplex.igenebook.cn

**Operating system(s):** User interface: Platform independent; Server side: Linux

**Programming language:** Flask, SQLite, HTML, jQuery, and Python

**Other requirements:** Web browser (supporting JavaScript)

**License:** None for usage

**Any restrictions to use by non-academics:** Licenses for the number of designed primer pairs for each target will be limited to 20 to save computational resources.

## Supplementary Information

**Additional file 1: Figure S1.** Amplification specificity and efficiency validation of single primer pairs. S refers to the human genome DNA sample, and N refers to the water negative control. Primer pair IDs are listed in Table S2.

**Additional file 2.**

**Additional file 3.**

**Additional file 4.**

**Additional file 5.**

**Additional file 6.**

## Data Availability

All data generated or analysed during this study are included in this published article and its Supplementary files, as well as in Genome Sequence Archive (https://ngdc.cncb.ac.cn/gsa-human/) under specific accession number (HRA001504). The Ultiplex multiplex primer design tool is available at http://ultiplex.igenebook.cn.The guidance video for this website is available at https://www.youtube.com/watch?v=Fm4b8yWyEgM.

## Abbreviations

PCR: Polymerase chain reaction
HNPCC: Hereditary nonpolyposis colorectal cancer
MMR: Mismatch repair gene
BLAST: The Basic Local Alignment Search Tool Tm Melting temperature
CPU: Central processing unit
