# Supplementary figures and images for: The web-based multiplex PCR primer design software Ultiplex and the associated experimental workflow: up to 100- plex multiplicity
Source: BMC Genomics. 2021 Nov 18;22:835. doi: 10.1186/s12864-021-08149-1 (PMC8600765; doi:10.1186/s12864-021-08149-1)

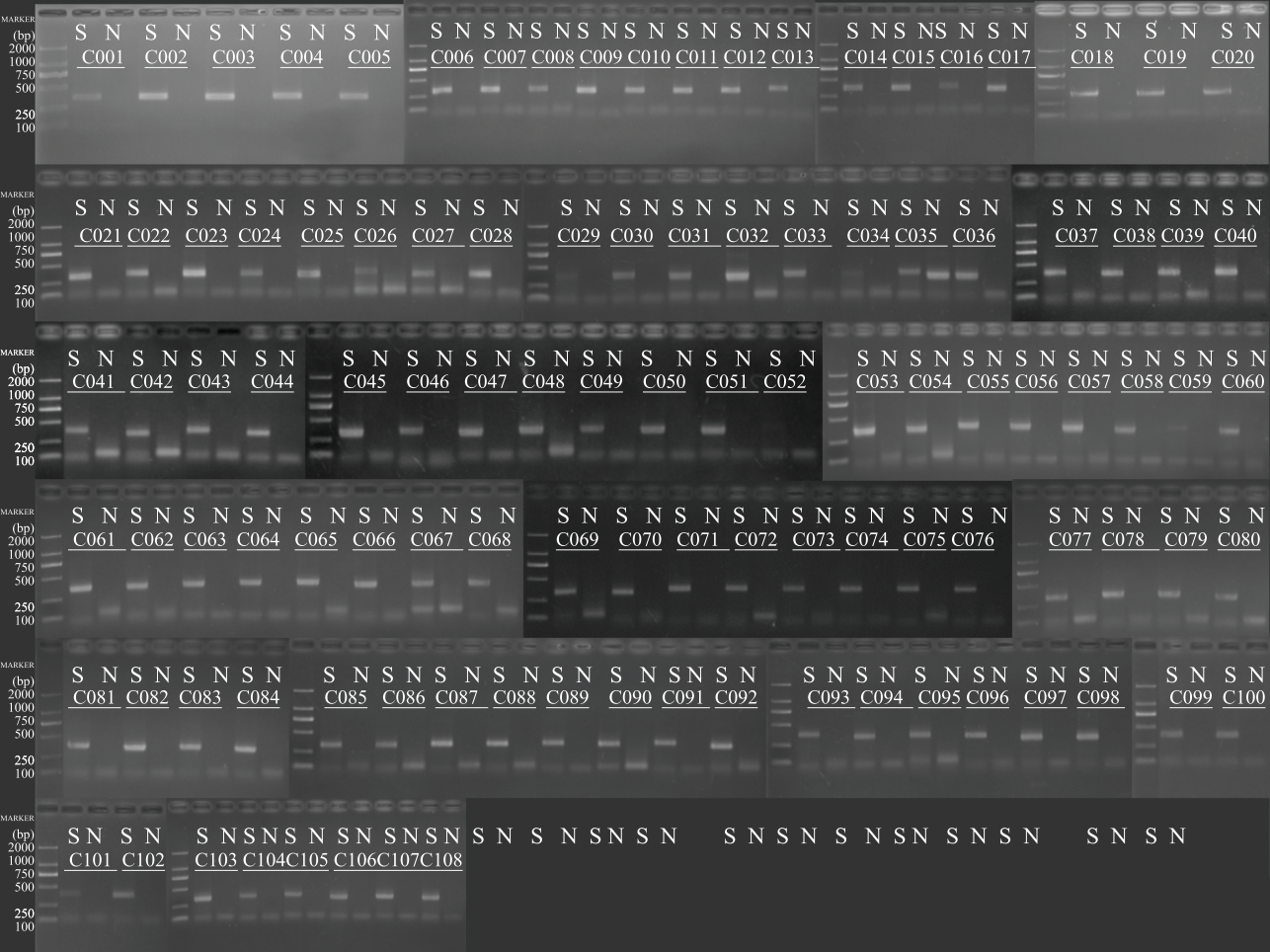

Supplement: Supplementary file 1 — Additional file 1: Figure S1. Amplification specificity and efficiency validation of single primer pairs. S refers to the human genome DNA sample, and N refers to the water negative control. Primer pair IDs are listed in Table S2. [file 12864_2021_8149_MOESM1_ESM.pdf]
